# Effectiveness of minimally invasive surgical procedures in the acceleration of tooth movement: a systematic review and meta-analysis

**DOI:** 10.1186/s40510-016-0146-9

**Published:** 2016-10-24

**Authors:** Alaa M. H. Alfawal, Mohammad Y. Hajeer, Mowaffak A. Ajaj, Omar Hamadah, Bassel Brad

**Affiliations:** 1 Orthodontics Department, University of Damascus Dental School, Damascus, Syria; 2 Oral Medicine Department, University of Damascus Dental School, Damascus, Syria; 3 Oral and Maxillofacial Surgery Department, University of Damascus Dental School, Damascus, Syria

**Keywords:** Orthodontics, Accelerated tooth movement, Minimally invasive, Surgical, Flapless, Corticotomy

## Abstract

**Objective:**

The objective of this study was to assess systematically the available scientific evidence relating the efficiency of minimally invasive surgical procedures in accelerating orthodontic tooth movement and the adverse effects associated with these procedures.

**Methods:**

Electronic search of these databases CENTRAL, EMBASE, Scopus, PubMed, Web of Science, Google Scholar Beta, Trip, OpenGrey and PQDT OPEN was performed (last updated January 2016). The reference lists of the included studies were hand searched. Unpublished literature and ongoing studies were also checked electronically through ClinicalTrials.gov and (ICTRP). Randomized controlled trials (RCTs) with patients who received minimally invasive surgical procedures combined with fixed orthodontic appliances compared with conventional treatment were included. Cochrane’s risk of bias tool was used to assess risk of bias.

**Results:**

Four RCTs (61 patients) and nine ongoing protocols were included in this review. Only three RCTs were suitable for quantitative synthesis. Higher tooth movement rate was found with the minimally invasive surgical procedures by a weighted mean difference of 0.65 mm for 1 month of canine retraction (WMD = 0.65: 95 % CI (0.54, 0.76), *p* < 0.001) and by a weighted mean difference 1.41 mm for 2 months (WMD = 1.41: 95 % CI (0.81, 2.01), *p* < 0.001). No adverse effects associated with these procedures were reported.

**Conclusions:**

There is limited available evidence about the effectiveness of minimally invasive surgically accelerated orthodontics (MISAO). Although the current review indicated that MISAO can help in accelerating canine retraction, further research in this domain should be performed before it can be recommended in everyday clinical practice.

**Electronic supplementary material:**

The online version of this article (doi:10.1186/s40510-016-0146-9) contains supplementary material, which is available to authorized users.

## Review

### Background

Comprehensive orthodontic treatment usually lasts for more than 1 year and a half when fixed appliances are used to treat moderate to severe cases of malocclusion [1], with a significant difference which can be affected by various factors [2, 3]. Increased orthodontic treatment duration has several adverse effects like pain, discomfort, caries, gingival recession and apical root resorption [4, 5]. Moreover, most adult patients want to finish their treatment at the earliest opportunity due to social and aesthetic concerns [6]. Therefore, both orthodontists and patients are interested in procedures that can accelerate tooth movement [7].

Recently, various methods have been suggested to reduce orthodontic treatment time such as adequate use of brackets, controlling force levels and relying on less friction bracket systems [8], photobiomodulation [9], pharmacological approaches [10] and low-intensity laser irradiation [11]. However, the most clinically applied and most examined with the potential of significantly decreasing treatment time are the surgical procedures [12]. Over the past, several forms of corticotomy has been used to reduce orthodontic treatment time [13, 14]. The acceleratory impact of corticotomies was attributed primarily to the so-called regional accelerated phenomena (RAP) [13, 15]. Furthermore, corticotomies may stimulate the expression of inflammatory markers and cytokines which leading to increased osteoclast activity [16–19].

Although corticotomy techniques have been effective in inducing rapid tooth movement [20–22], they were relatively invasive requiring full mucoperiosteal flaps, suturing in conjunction with the associated surgical risks such as pain, swelling [23], slight interdental bone and attached gingiva loss [24]. This may explain the lack of their common spread among orthodontists in their daily practice. So as a result, more conservative flapless corticotomy techniques have been proposed in the last years, such as corticision [25], piezocision [26–28], micro-osteoperforations (MOPs) [17, 29], and laser-assisted flapless corticotomy [30, 31].

It has been claimed that orthodontic treatment advances faster with negligible danger of side effects after minimally invasive surgical procedures, but there is a lack of the evidence related to the effectiveness of minimally invasive surgical procedures in accelerating tooth movement. Thus, the aim of this review is the critical and systematic appraisal of the available evidence regarding the effectiveness of minimally invasive surgical procedures in inducing rapid orthodontic tooth movement and the untoward effects of these surgical interventions.

### Materials and methods

Primarily, a PubMed pilot search was performed, and then, two potentially eligible trials were assessed before writing the protocol. Registration with PROSPERO was performed during the first stages of this review (http://www.crd.york.ac.uk/PROSPERO/display_record.asp?ID=CRD42016036737; 2016: CRD42016036737). This systematic review was written according to the Cochrane Handbook for Systematic Reviews of Interventions Version 5.1.0 [32] and the Preferred Reporting Items for Systematic Reviews and Meta-Analyses (PRISMA) guidelines [33, 34].

#### Eligibility criteria

Criteria of exclusion and inclusion were employed with reference to the Participants, Interventions, Comparisons, Outcomes and Study design (PICOS) framework. *Study design*: in vivo randomized controlled trials (RCTs) without any restrictions on publication year or language were included. *Participants*: healthy patients, both males and females with any age and type of malocclusion, of any ethnic group who received orthodontic treatment with fixed orthodontic appliances were included. *Type of interventions*: any sort of orthodontic treatment with fixed appliances (with/out the need to extract teeth in the context of treatment) assisted by minimally invasive surgical techniques for accelerating orthodontic tooth movement (i.e. corticision, piezocision, micro-osteoperforations, laser-assisted flapless corticotomy, interspetal bone reduction or any surgical procedure which is not required raising flap) were included. *Comparisons*: patients receiving conventional orthodontic treatment with fixed appliances (without any additional intervention to accelerate tooth movement). *Outcomes*: primary outcome: the rate of tooth movement (RTM) or any equivalent measurement that would give an idea about the efficacy of the non-invasive surgical procedure. Secondary outcomes: adverse side effects such as patient-reported outcomes (pain, discomfort, oral-health-related quality of life, alteration in mastication, other experiences and satisfaction), or gingival and periodontal complications including (gingival recession, loss of attachment, depth of probing, bone resorption), or loss of anchorage and unwanted tooth movement (tipping, torquing, rotation) or iatrogenic harm to teeth (e.g., tooth vitality loss, root resorption) or stability of treatment in the long term.

#### Search strategy

Electronic search was performed in January 2016 with no time and language limitations in the following databases: The Cochrane Central Register of Controlled Trials (CENTRAL), EMBASE, Scopus, PubMed, Web of Science, Google Scholar Beta, Trip, OpenGrey (to identify the grey literature) and PQDT OPEN from proQuest (to identify dissertations and theses). Details of the electronic search strategy are provided in Additional file 1: Table S1. The reference lists of selected papers and relevant reviews were screened for any possible related studies which may have not been discovered by the electronic web-based search. ClinicalTrials.gov and World Health Organization International Clinical Trials Registry Platform Search Portal (ICTRP) were also checked electronically to retrieve any unpublished studies or currently accomplished research work (Additional file 2: Table S2).

#### Study selection and data extraction

Two reviewers (AMHA and MYH) assessed independently eligibility of the trials, and in case of disagreement, a third author (MAA) was asked to resolve this. The first check included only titles and abstracts. Full-text assessment was the second step for all papers appearing to be relevant and candidate for inclusion or when the title or abstract was vague to help in reaching an obvious judgement. Papers were excluded when they did not fulfil one or more of the inclusion criteria. Corresponding authors were e-mailed for obtaining clarifications or extra data.

The same two authors (AMHA and MYH) conducted data extraction independently in the piloted and pre-defined data extraction tables. A third author (OH) was consulted when there was a disagreement between the two authors to arrive at a resolution. The data extraction sheet included the following items: general information (the name of authors, the year of publication and study setting); methods (study design, treatment comparison); participants (sample size, age, gender); intervention (type of interventions, intervention site, technical aspects of interventions); orthodontic aspects (malocclusion characteristics, type of movement, appliance characteristics and biomechanics, frequency of orthodontic adjustments, follow-up time) and outcomes (primary and secondary outcomes mentioned, methods of outcome measurements, statistical significance of reported difference treated vs. controls).

#### Assessment of risk of bias in included studies

The quality of the included studies was evaluated by two reviewers (AMHA and MYH) using Cochrane’s risk of bias tool [35]*.* When lack of consistency was observed, a third author (MAA) was consulted to arrive at a resolution.

We evaluated the following fields as at low, high or unclear risk of bias: sequence generation (selection bias), allocation concealment (selection bias), blinding of participants and personnel (performance bias), blinding of outcome assessors (detection bias), incomplete outcome data addressed (attrition bias), selective outcome reporting (reporting bias) and other bias.

The overall risk of bias of the included trials was assessed according to the following: *low risk of bias*: if all fields were evaluated as at low risk of bias (bias improbable to change the results critically), *unclear risk of bias*: if at least one or more fields were assessed as at unclear risk of bias (bias carries some doubt about the results) and *high risk of bias*: if at least one or more fields were evaluated as at high risk of bias (bias affect the results critically) (excluded from the primary analysis).

#### Data synthesis and statistical analysis

To evaluate the amount of heterogeneity of the included trials, the treatment interventions, treatment protocols, place of research, patients, methodology and outcome measures were assessed. From the statistical point of view, heterogeneity was first evaluated visually and then mathematically. Two reviewers (AMHA. and MYH.) checked the graphical display of the estimated treatment effects with 95 % confidence intervals. The *I* ^2^ index was calculated to assess heterogeneity, and its values were interpreted as follows: *low heterogeneity*: 0 to 40 %, *moderate to high heterogeneity*: 30 to 60 %, *significant heterogeneity*: 50 to 90 % and *very significant heterogeneity*: 75 to 100 %. Significant heterogeneity was also considered when *p* values were less than 0.1 when applying *χ* ^2^ tests [36]. Data was pooled to meta-analysis when trials had comparable interventions, subjects and outcomes. Mean differences (MD) with their associated 95 % confidence intervals (CI) were chosen to express results as effect measure. The treatment effect was weighted (weighted mean difference ( WMD)) using calculations based on a random effects model.

This model was deemed suitable because of the observed differences in settings and populations. The inverse-variance method was chosen in cases of split-mouth design, and the standard deviation (SD) of the difference was calculated according to this formula:

$$ \sqrt{\mathrm{Sd}{1}^2+\mathrm{S}\mathrm{d}{2}^2-2 \times r \times \mathrm{S}\mathrm{d}1 \times \mathrm{S}\mathrm{d}2\ } $$

where Sd1 and Sd2 are the standard deviations between quadrants, respectively, and *r* is the correlation coefficient between quadrants. We deemed *r* = 0.5 for split-mouth designs and *r* = 1 for parallel designs. Then, the standard error (SE) was calculated: SE = SD/√(*n*). Meta-analyses were undertaken in (Review Manager (RevMan), Version 5.3. Copenhagen: The Nordic Cochrane Centre, the Cochrane Collaboration, 2014).

In a future update of this review, if an appropriate number of papers (i.e. more than 10 papers) evaluated identical interventions and they have been included in a meta-analysis, publication bias will be evaluated using standard funnel plots. In addition, subgroup analyses based on the type of interventions (piezocision, micro-osteoperforations, interseptal bone reduction, corticision, lasercision) and age stage (adolescents, adults) will be done. Subgroup analysis is important especially when there is considerable heterogeneity.

If an adequate number of trials are included in future updates, the vigour of the results will be evaluated using sensitivity analyses. This will include repeating the analyses after eliminating high-risk bias trials or studies with high chance of heterogeneity involving dominant effects of large studies and the variation in outcomes related to the scenario of orthodontic treatment (extraction, non-extraction), or type of minimally invasive surgery (piezocision, micro-osteoperforations, interseptal bone reduction), or age stage (adolescents, adults) to isolate their impact on the results.

The overall quality of evidence was evaluated according to Grading of Recommendations Assessment, Development and Evaluation (GRADE) approach using summary of findings table [37].

### Results

#### Literature flow

**Fig. 1:**
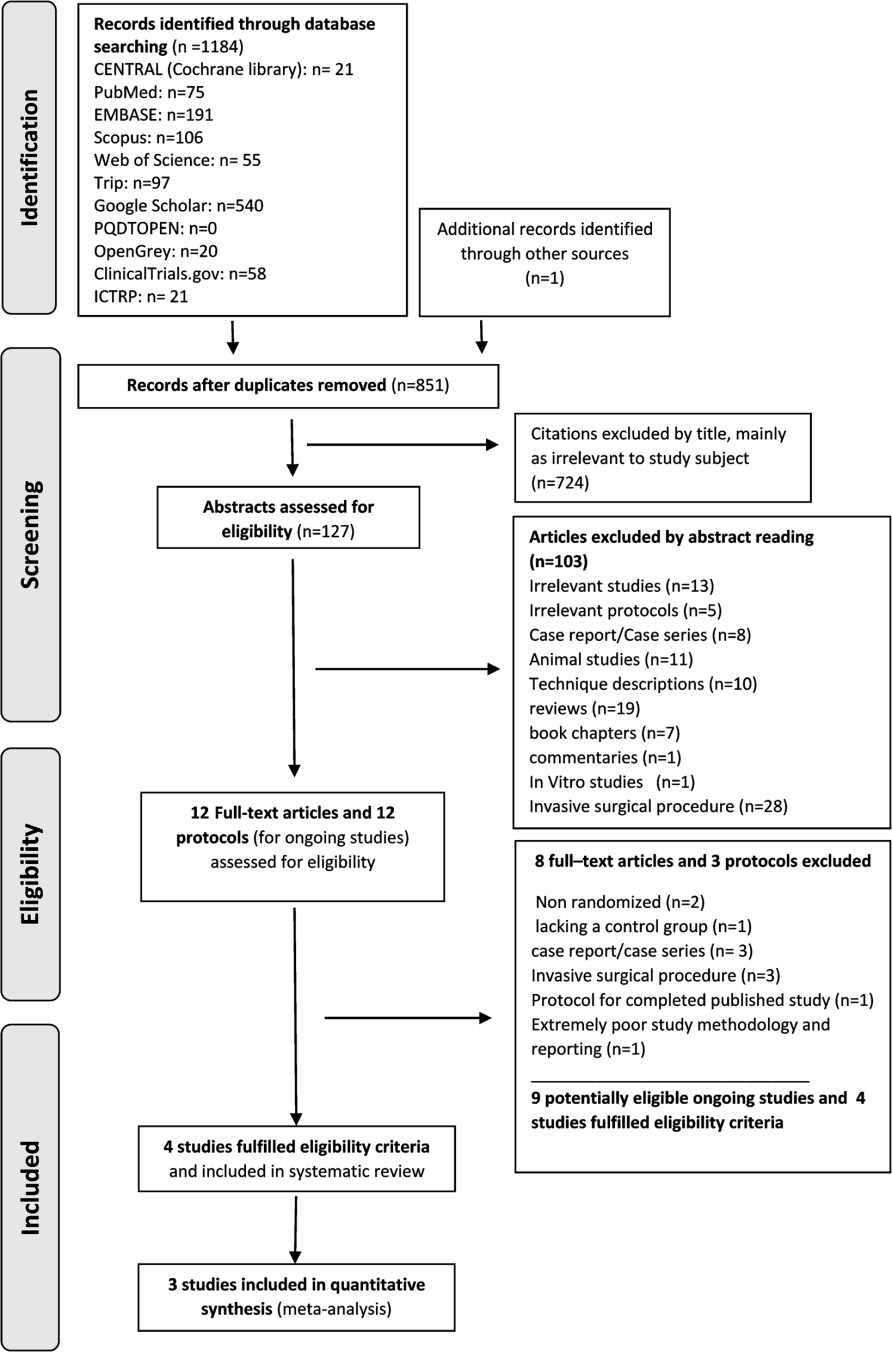
PRISMA 2009 flow diagram of the included studies

One thousand one hundred eighty-four references were found in the electronic search, and only one more citation was identified from other sources. Duplicate references were taken off, and a total of 851 citations were carefully checked. The titles and abstracts were screened for eligibility, and then, all papers which were not fulfilling the inclusion criteria were eliminated. As a result, 24 potentially related trials (12 studies and 12 registry entries for ongoing research work) were examined in depth. Eight of the completed studies and 3 of the ongoing studies were eliminated after full-text reading of the papers (Additional file 3: Table S3: the excluded trials and the reasons for exclusion). Finally, we had 4 studies and 9 ongoing studies to include. The PRISMA flow diagram is shown in Fig. 1.

#### Characteristics of studies

The characteristics of the four included trials [17, 38–40] can be found in Table 1 and Additional file 4: Table S4. The nine included protocols were for RCTs trials; more information about these ongoing research projects are given in Table 2 and Additional file 5: Table S5.

**Table 1:** Characteristics of included studies in the systematic review

Study/setting	Methods		Participants	Interventions		Outcomes
	Study design	Treatment comparison	Patients (M/F) Mean age (years) Malocclusion	Type and site of intervention/technical aspects of interventions	Follow-up time	Primary and secondary outcomes
Alikhani 2013 [17] New York USA	RCT COMP	MOP + OT vs. OT	Patients (M/F): 20 (8/12) Control: 10, Exp: 10 Mean age: Control: 24, 7 Exp:26, 8 Malocclusion: class II div.1	- MOPs (upper canines) - No flap elevation, three small MOPs were done in the extraction area at equivalent spaces between the canine and the second premolar after 6 months from maxillary first premolar extraction. Each perforation was 1.5 mm wide and 2 to 3 mm deep. Surgical instrument: a disposable handled device^a^ orthodontic activation: immediately following the intervention	4 weeks	Primary outcome: RTM (mm/month) Secondary outcomes: -Pain and discomfort -Inflammatory markers (cytokines levels)
Mehr 2013 [38] Connecticut USA	RCT (PG)	Piezocision + OT vs. OT	Patients (M/F): 13 (5/8) Control: 6 Exp: 7 Mean age (years): Control: 26, 35 Exp: 29, 12 Malocclusion: mandibular anterior crowding (irregularity index greater than 5)	- Piezocision (mandibular incisors) - No flap elevation, three vertical incisions, (4 mm length and 1 mm depth of cortical bone), interproximally between mandibular canines and lateral incisors, and central incisors. Surgical instrument: piezosurgery knife (BS1) orthodontic activation: immediately following the intervention	Until complete decrowding	Primary outcome: -RTM (mm/month) -TTM (days) Secondary outcomes: pain
Leethanakul 2014 [39] Thailand	RCT (SP)	Interseptal bone reduction + OT vs. OT	Patients (M/F): 18 (0/18( Control: 18, Exp: 18 Mean age (years): 21.9 ± 4.7 Malocclusion: patients who need to extract maxillary 1st premolars and maxillary canine retraction	- Interseptal bone reduction (upper canines) - No flap elevation, reduction (1.0 to 1.5 mm) of the interseptal bone distal to the canine inside the extraction socket of the first premolar. Surgical instrument: bur orthodontic activation: immediately following the intervention	Up to 3 months after intervention	Primary outcome: -RTM (mm/month) -CTM (mm) Secondary outcomes: Canine tipping Canine rotation
Aksakalli 2015 [40] Istanbul Turkey	RCT (SP)	Piezocision + OT vs. OT	Patients (M/F): 10 (4/6) Control: 10, Exp: 10 Mean age (years): 16.3 ± 2.4 (adult only) Malocclusion: half or more unit class II malocclusion	- Piezocision (upper canines) - No flap elevation, two vertical interproximal incisions were performed mesial and distal of the maxillary canines, 5 mm apical to interdental papilla, incision lengths were approximately 10 mm apically, 3 mm deep in cortical alveolar. Surgical instrument: piezosurgery knife (BS1) orthodontic activation: immediately following the intervention.	Up to ideal class I canine relation-ship	Primary outcome: -CTM (mm) -TTM (months) Secondary outcomes: - Molar anchorage loss - Transversal changes - Mobility scores - Gingival indices

*RCT* randomized clinical trial, *OT* orthodontic therapy, *PG* parallel-group design, *SP* split-mouth design, *COMP* compound design (parallel-group design and one arm is a split-mouth design), *MOPs* micro-osteoperforations, *Exp* experimental, *NR* not reported, *M* male, *F* female, *U3* upper canines, *SS* stainless steel, *RTM* rate of tooth movement, *TTM* time of tooth movement, *CTM* cumulative tooth movement

^a^PROPEL orthodontics, Ossining, NY

**Table 2:** Protocols of the ongoing studies registered at the clinical.trials.gov and the ANZCTR

Study ID	Trial name or title	Study design	Intervention + treatment comparison	Sample size/age/gender	Outcomes
NCT02606331	Efficacy of minimally invasive surgical technique in accelerating orthodontic treatment	-RCT/PG -Single blind (outcomes assessor)	-Piezocision + OT vs. OT - MOPs accomplished by ER: YAG laser + OT vs. OT	36/15–27/both (male, females)	Primary outcomes: rate of canine retraction Secondary outcomes: rate of molar anchorage loss/canine rotation/levels of pain and discomfort
NCT02359760	Assessment of piezoelectric periodontal surgery effects on orthodontic treatment: a prospective pilot study	- RCT/PG -Single blind (outcomes assessor)	Piezocision + OT vs. OT	EXP:20, CON:40/18–40/both (male, females)	Primary outcomes: duration of orthodontic treatment Secondary outcomes: compare the duration of treatment with the control group/overall quality of treatment (ABO) standards/pain/inflammatory markers/density and bone volume/root resorption
NCT02590835	Efficiency of piezocision-assisted orthodontic treatment in adult patients	- RCT/PG -Open label	Piezocision + OT vs. OT	24/21 years and older/both (male, females)	Primary outcomes: overall treatment time measurement. Secondary outcomes: root resorption/periodontal parameters/patient-centred outcomes
NCT01720797	Alveolar micro-perforation for inflammation-enhanced tooth movement during orthodontic treatment (propel)	- RCT/PG - Open label	MOPs + OT vs. OT	15/18–55/both (male, females)	Primary outcomes: tooth movement Secondary outcomes: NR
NCT02549950	Efficiency of piezo-corticision in accelerating orthodontic tooth movement	- RCT/PG - Open label	Peizo-corticision + OT vs. OT	NR/15–35/both (male, females)	Primary outcomes: rate of orthodontic canine movement. Secondary outcomes: rate of orthodontic incisor retraction/quality of treatment outcome (ABO) standards
NCT02473471	Micro-osteoperforation and tooth movement	- RCT/PG -Double blind (investigator outcomes assessor)	MOPs + OT vs. OT	40/13–45/both (males, females)	Primary outcomes: rate of tooth movement Secondary outcomes: pain/root resorption/patient satisfaction
NCT02571348	Optimum micro-osteoperforations accelerated tooth movement interval	-RCT/PG -Single blind (outcomes assessor)	MOPs + OT vs. OT	36/18–45/both (males, females)	Primary outcomes: rate of orthodontic tooth movement Secondary outcomes: rate of orthodontic tooth movement between maxilla and mandible and when micro-osteoperforations performed at 4-, 8- and 12-week intervals/pain.
NCT02416297	Three-dimensional evaluation of accelerated tooth movement	- RCT/PG - Open label	MOPs + OT vs. OT	50/16–60/both (males, females)	Primary outcomes: velocity rate of anterior retraction/bone demineralization Secondary outcomes: NR
ACTRN12615000593538 Register: ANZCTR	The effects of micro-osteoperforations on orthodontic root resorption and tooth movement—a pilot study	-RCT/SP -Double blind (investigator, outcomes assessor)	MOPs + OT vs. OT	15/12–18/both (males, Females)	Primary outcomes: root resorption Secondary outcomes: orthodontic tooth movement/level of discomfort

*RCT* randomized clinical trial, *PG* parallel-group design, *SP* split-mouth design, *MOPs* micro-osteoperforations, *OT* orthodontic therapy, *NR* not reported, *EXP* experimental group, *CON* control group, *ABO* American Board of Orthodontics’ grading system

Minimally invasive surgical procedures without flap raising or suturing were undertaken in all included studies [17, 38–40]. Two studies evaluated piezocision [38, 40], one tested micro-osteoperforation [17] and one trial investigated the effect of interseptal bone reduction [39]. Sixty-one adult participants 44 female and 17 male were included in the four trials. Extraction treatment (upper canine retraction) was performed in three papers [14, 39, 40], and only one study was a non-extraction study with mandibular anterior crowding [38]. From the nine protocols for ongoing studies, five investigated the effect of micro-osteoperforations, three tested piezocision and one evaluated both piezocision and micro-osteoperforation; only two were labelled as ‘completed studies’.

#### Risk of bias of included studies

**Fig. 2:**
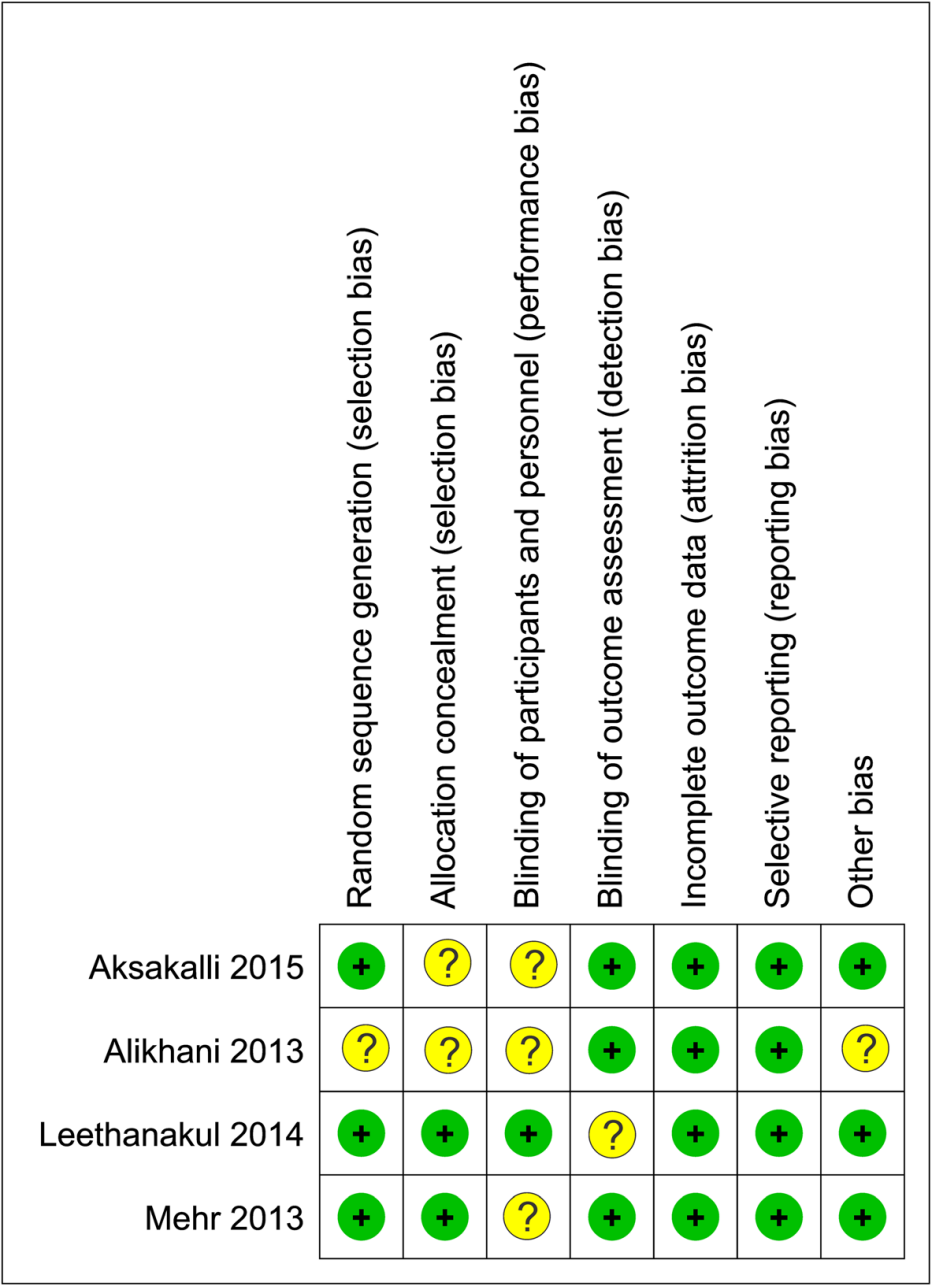
Risk of bias summary of RCTs. Low risk of bias (the *plus sign*); unclear risk of bias (the *question mark sign*)

**Fig. 3:**
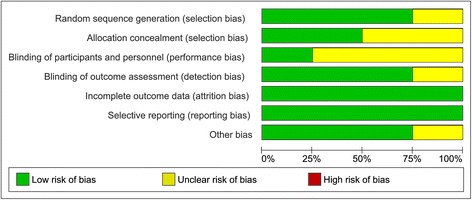
Overall risk of bias score for each field

Figures 2 and 3 show the summary of the overall risk of bias of the included studies. ‘Unclear risk of bias’ was the common feature between the four studies. Concealment of allocation as well as participants’ blinding were the most problematic fields (unclear in 50 %, 75 % of studies, respectively). Further details of the assessment of risk of bias can be found in Additional file 6: Table S6.

#### Effects of interventions

##### Primary outcome (rate of tooth movement)

Three from the four included trials assessed the effect of minimally invasive corticotomy on the rate of canine retraction after premolar extraction and were considered appropriate for quantitative synthesis [17, 39, 40]; the fourth trial [38] evaluated the effect of piezocision on the rate of alignment in a non-extraction treatment. Therefore, the results of this study could not be pooled to a meta-analysis with the other trials due to the variation in outcomes related to the scenario of orthodontic treatment (extraction, non-extraction).

#### Canine retraction amount at 1 month

**Fig. 4:**
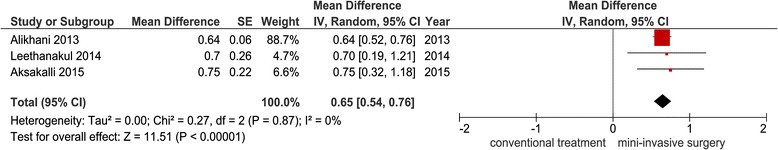
Forest plot of the comparison between minimally invasive surgical procedures and conventional treatment for the canine retraction amount at 1 month

Three trials compared the cumulative tooth movement of upper canines after the first premolar extraction between conventional and minimally invasive surgical methods after 1 month [17, 39, 40]. The pooled estimate suggested a greater tooth movement by 0.65 mm with minimally invasive surgical procedures compared to the conventional procedure for the first month of canine retraction, a statistically significant finding (Fig. 4; WMD = 0.65; 95 % CI (0.54, 0.76); *p* < 0.001). Heterogeneity was low (*χ* ^2^ = 0.27, *p* = 0.87, *I* ^2^ = 0 %). The overall quality of evidence for canine retraction rate at 1 month is very low according to GRADE (Table 3).

**Table 3:** Summary of findings table according to the GRADE guidelines for the included trials

Patient or population: patients need orthodontic treatments, settings: upper canines (RCT), intervention: minimally invasive surgical procedures, comparison: conventional treatment				
Outcomes	Weighted mean difference (95 % CI) between minimally invasive surgical assisted vs. conventional retraction	No. of participants (studies)	Quality of the evidence (GRADE)	Comments
Orthodontic tooth movement in mm (2 months)	The mean canine retraction in the intervention groups was 1.41 higher (0.81 to 2.01 higher). Relative effect (95 % CI): not estimable	28 (2 studies SP)	⊕⊕⊝⊝^a^ Low	This outcome also measured at 1 month in 3 studies (38 patients): mean canine retraction in the intervention groups was 0.65 higher (0.54 to 0.76 higher) with a quality of evidence very low ⊕⊝⊝⊝^b^. Also, this outcome was assessed at 3 months in one study (18 patients). This study reported higher tooth movement by 2 mm with the minimally invasive surgical procedure, a statistically significant finding (MD = 2: 95 % CI 1.20 to 2.80) with a quality of evidence very low ⊕⊝⊝⊝^c^.
Pain and discomfort	See comments Relative effect (95 % CI): not estimable	10 (1 study, COMP)	⊕⊝⊝⊝^d^ Very low	The difference between the control and experimental groups was not significant (*p* > 0.5) at 1, 7, 14 and 28 days after retraction.
		13 (1 study, PG)	⊕⊕⊝⊝^e^ Low	There was no significant difference in the level of pain between the two groups immediately, 1 and 12 h and 7 days after piezocision (*p* > 0.05). We could not pool the results of the previous 2 trials which evaluated this outcome to quantitative synthesis due to differences in specific treatments (non-extraction vs. extraction).
Adverse effects (periodontal problems)	See comments Relative effect (95 % CI): not estimable	10 (1 study, SP)	⊕⊕⊝⊝^f^ Low	There was no significant difference in mobility scores of canines between the control and experimental groups pre- and post-distalization (*p* > 0.05). Similarly, there was no significant difference in gingival indices between both groups pre- and post-distalization (*p* > 0.05).
Adverse effects anchorage loss	See comments Relative effect (95 % CI): not estimable	10 (1 study, SP)	⊕⊕⊝⊝^f^ Low	There was significant difference in loss of anchorage between control and experimental groups (*p* < 0.05), the anchorage loss was lesser in the piezocision group.
Adverse effects (unwanted tooth movement)	See comments Relative effect (95 % CI): not estimable	18 (1 study, SP)	⊕⊕⊝⊝^g^ Low	There was no significant difference between control and experimental sides for canine tipping and rotation (*p* > 0.05).
		10 (1 study, SP)	⊕⊕⊝⊝^f^ Low	There was no significant difference between control and experimental sides for transversal changes (*p* > 0.05).

High quality: further research is very unlikely to change our confidence in the estimate of effect. Moderate quality: further research is likely to have an important impact on our confidence in the estimate of effect and may change the estimate. Low quality: further research is very likely to have an important impact on our confidence in the estimate of effect and is likely to change the estimate. Very low quality: we are very uncertain about the estimate

*CI* confidence interval, *PG* parallel-group design, *SP* split-mouth design, *COMP* compound design, *GRADE* working group grades of evidence

^a^Decline one level for risk of bias (blinding of outcome assessment unclear [39], blinding of participants and personnel and allocation concealment unclear in [40]) and one level for indirectness*

^b^Decline two levels for risk of bias (blinding of participants and personnel and allocation concealment unclear [17, 40], blinding of outcome assessment unclear [39]), random sequence generation and bias due to conflict of interest unclear [17]) and one level for indirectness*

^c^Decline one level for risk of bias (blinding of outcome assessment unclear [39]), and one level for indirectness*, and one level for imprecision**

^d^Decline two levels for risk of bias (unclear risk of bias of randomization, allocation concealment, blinding of participants and personnel and conflict of interest [17]) and one level for imprecision**

^e^Decline one level for risk of bias (unclear risk of bias of blinding of participants and personnel [17]) and one level for imprecision**

^f^Decline one level for risk of bias (unclear risk of bias of blinding of participants and personnel and allocation concealment [40]) and one level for imprecision**

^g^Decline one level for risk of bias (unclear risk of bias of blinding of outcome assessment [39]) and one level for imprecision**

*Outcome is not directly related; the included trials involved only adult patients, so the efficacy of minimally invasive surgical procedures could not be confirmed on adolescent patients. Also, patient-centred outcomes were very limited

**Limited number of trials, of limited sample size

#### Canine retraction amount at 2 months

**Fig. 5:**
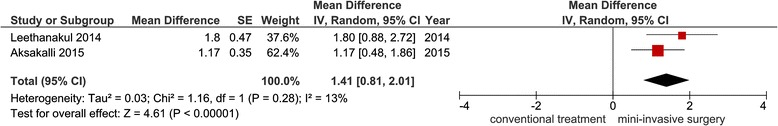
Forest plot of the comparison between minimally invasive surgical procedures and conventional treatment for the canine retraction amount at 2 months

Two trials only compared the cumulative tooth movement of upper canines after the first premolar extraction between conventional and minimally invasive surgical methods after an observation period of 2 months [39, 40]. The pooled estimate suggested a greater tooth movement of about 1.41 mm with minimally invasive surgical procedures compared to the conventional procedure for 2 months of canine retraction, a statistically significant finding (Fig. 5; WMD = 1.41; 95 % CI (0.81, 2.01); *p* < 0.001). Heterogeneity was low (*χ* ^2^ = 1.16, *p* = 0.28, *I* ^2^ = 13 %). The overall quality of evidence supporting this outcome is low according to GRADE (Table 3).

#### Canine retraction amount at 3 months

Only one trial assessed the cumulative tooth movement of upper canines after the first premolar extraction between conventional and minimally invasive surgical methods after an observation period of 3 months [39]. This study reported a greater tooth movement of about 2 mm with the minimally invasive surgical procedures compared to the conventional procedure, a statistically significant finding (MD = 2; 95 % CI (1.20, 2.80)). The overall quality of evidence supporting this outcome is very low according to GRADE (Table 3).

The trial of Mehr evaluated the influence effect of piezocision on the treatment time required for the relief of lower anterior crowding [38]. This study reported no significant difference in treatment time between conventional and experimental group (118.40 vs. 98.50 days, respectively, *p* = 0.43; MD = −19.90, 95 % CI (−59.53, 19.73)(, whereas the rate of alignment was slightly higher in the experimental compared to control group only during the first month (*p* = 0.035; MD = −0.04: 95 % CI (−0.07, −0.01)).

##### Secondary outcomes

Two trials evaluated the levels of pain associated with minimally invasive surgical procedures [17, 38]; we could not pool the results to quantitative synthesis due to the difference between the two studies in the provided treatment (extraction vs. non-extraction, retraction vs. relief of crowding). Alikhani assessed pain and discomfort levels during canine retraction using numeric rating scale, and there was no significant difference between the control and experimental sides (*p* > 0.5) at 1, 7, 14 and 28 days after retraction [17]. A visual analogue scale was used by Mehr for the assessment of pain when acceleration of tooth decrowding was under inspection, and there was no significant difference between the control and experimental groups immediately, 1 and 12 h and 7 days after the piezo-surgical intervention (*p* > 0.05) [38].

Gingival and periodontal problems, including gingival inflammation and mobility for the retracted canines, were evaluated in a one split-mouth design study [40]. There was no significant difference in the mobility scores between the control and the experimental sides pre- and post-distalization (*p* > 0.05). Similarly, there was no significant difference in gingival indices between both sides pre and post-distalization (*p* > 0.05).

Undesirable posterior and anterior tooth movements were inspected in two studies. Aksakalli investigated molar anchorage loss and transversal changes of the upper canines and first upper molars. There was a significant decrease in loss of anchorage in the piezocision side compared to the control side (*p* < 0.05). For transversal measurements related to the distance from the mid-palatal suture to the upper canine/first molar, there was no significant difference between the two sides (*p* > 0.05) [40]. On the other hand, for the untoward side effects following retraction of anterior teeth, Leethanakul studied both canine tipping and rotation and there were no significant differences between the control and the experimental sides (*p* > 0.05) [39].

### Discussion

Minimally invasive surgically accelerated orthodontics (MISAO) has captured the attention of the orthodontic community in recent years. The increased number of registered clinical trials in this domain has reflected the great interest in this topic. According to this systematic review, it appears that there is a low evidence showing a significant advantage of the minimally invasive surgical procedures relative to the possible associated adverse effects. We included only the randomized controlled trials to minimize bias and possible confounders. MISAO has been evaluated in four trials which were judged to be at unclear risk of bias; this could have potentially confused the results.

Three trials investigated the efficacy of minimally invasive surgical procedures in accelerating canine retraction after premolars extraction [17, 39, 40], and they reported a greater tooth movement with the minimally invasive surgical procedures compared to the conventional method by 0.65 and 1.41 mm for the first and second months, respectively. However, the effectiveness of these procedures is doubtful over time since the impact on the overall treatment time was not investigated. Whereas only one included study evaluated the effectiveness of minimally invasive surgical procedures in accelerating alignment (non-extraction treatment) and reported no significant difference in the overall treatment time between the conventional and the experimental groups [38]. When comparing the findings of this non-extraction-based trial [38] with the three previous extraction-based studies [17, 39, 40], it seems that the treatment type could have an influence on the accelerating rate. An explanation for this may be due to the difference in the type of movement (rotation, tipping, bodily movement) or due to the application site or bone density (mandibular vs. maxillary arch).

The previous included studies assessed different minimally invasive surgical procedures piezocision [38, 40], MOPs [17] and interseptal bone reduction [39] with variations on the size and design of the flapless corticotomy cuts; in addition, there was a variation on the used tools to perform these procedures, such as piezosurgical tools [38, 40], disposable perforating devices [17] and lasers [30]. Therefore, it is important that future studies test the effect of these differences in (surgical protocol, size and design of cuts, tools) on the amount of accelerating, treatment costs and the adverse side effects of each individual intervention.

Sixty-one participants were included in the four studies, and this number is relatively small. More prospective RCTs with increased sample size are required. The four trials included only adult patients; therefore, the effectiveness of these procedures in adolescent patients was not confirmed.

Gender can be considered a confounding factor when analysing bone remodelling and the rate of tooth movement. However, the effect of gender was not isolated in this review because of the small number of studies included as well as the small sample sizes employed.

Moreover, the reduction of the tooth movement rate is apparent after the first month of application [17] or after the second month [39]. One possible explanation is the transient nature of the RAP which is manifested by a decline in the tooth movement rate over time [13, 15]. Therefore, the possible advantage of these minimally invasive procedures might be weakened over the time making it of little value at the final assessment of outcome. Therefore, future research should evaluate the effect of repeated interventions on decreasing overall treatment time as well as the possible untoward effects on the periodontal tissues.

No adverse effects of MISAO on periodontal tissues were reported in the included trials [40]. However, the assessment did not take into account important variables such as plaque index, probing depth, attachment loss and gingival recession.

Two included trials investigated the unwanted tooth movements (canine tipping and rotation, molar anchorage loss, transverse changes) associated with MISAO [39, 40]. No significant unwanted tooth movements were reported; conversely, Aksakalli found less loss of molar anchorage in the piezocision side compared to the control side [40]. This may be explained by the possible localized alveolar response in the injury area which did not extend backwards to the posterior segments.

No significant levels of pain and discomfort associated with MISAO were reported [17, 38]. This can be attributed to the conservative nature of these surgical interventions since no flap reflection or suturing was required.

Although no important adverse effects associated with MISAO were reported in the evaluated four trials, there is no scientific evidence to support the absence or presence of post-operative infection, bleeding, swelling, root resorption, loss of tooth vitality and possible morbidity following such procedures. It is therefore suggested that future work should consider these effects and patient-reported outcomes. Although a meticulous approach was employed in the current review to retrieve the relevant papers that would answer the focused review question, there are still some limitations due to the variability between the included trials in fixed appliance characteristics, biomechanics of tooth movement, and the methods of outcome measurements.

## Conclusions

There is limited and low-quality evidence concerning the efficacy of MISAO in acceleration orthodontic tooth movement. Therefore, the results of this systematic review should be taken carefully, and there is a need for more research with additional attention paid to sample size, overall treatment follow-up, the applied surgical protocol (extent, size and design of surgical cuts), the type of the minimally invasive techniques (piezocision, micro-osteoperforations, interseptal bone reduction, corticision, lasercision), the type of orthodontic treatments (extraction vs. non-extraction), the number of surgical interventions (single vs. repeated interventions), adverse effects and cost-benefit ratio. MISAO cannot currently be recommended in everyday clinical practice, although the acceleration of canine retraction appeared to be significant at least in the first 2 months according to this review.

## Additional files

Additional file 1: Table S1. Electronic Search Strategy. (DOCX 19 kb)
Additional file 2: Table S2. Search strategy for trials’ registries. (DOCX 13 kb)
Additional file 3: Table S3. Excluded studies and the reasons beyond exclusion. (DOCX 16 kb)
Additional file 4: Table S4. (Supplemental to Table 1): Additional Characteristics of the included studies. (DOCX 16 kb)
Additional file 5: Table S5. (Supplementary to Table 2): Additional Characteristics of the protocols (ongoing studies). (DOCX 15 kb)
Additional file 6: Table S6. Risk of bias. (DOCX 16 kb)

## Abbreviations

CCTs: Controlled clinical trials
CENTRAL: The Cochrane Central Register of Controlled Trials
CI: Confidence intervals
CTM: Cumulative distance of tooth movement
GRADE: Grading of Recommendations Assessment Development and Evaluation
MD: Mean differences
MISAO: Minimally invasive surgically accelerated orthodontics
MOPs: Micro-osteoperforations
PRISMA: Preferred Reporting Items for Systematic Reviews and Meta-Analyses
r: Correlation coefficient
RAP: Regional accelerated phenomena
RCTs: Randomized controlled trials
RTM: Rate of tooth movement
SD: Standard deviation
SE: Standard error
WMD: Weighted mean difference
