# Supplementary material for: Effectiveness of minimally invasive surgical procedures in the acceleration of tooth movement: a systematic review and meta-analysis
Source: Prog Orthod. 2016 Oct 24;17:33. doi: 10.1186/s40510-016-0146-9 (PMC5075528; doi:10.1186/s40510-016-0146-9)
Supplement: Additional file 1: Table S1. — Electronic Search Strategy. (DOCX 19 kb) [file 40510_2016_146_MOESM1_ESM.docx]

| **Table S1: Electronic Search Strategy** | |
| --- | --- |
| **Database** | **Search Strategy** |
| **CENTRAL (The Cochrane Library)**  **Issues: 1 of 12  Publication Date: from no limit Until 18-1-2016 Search field:  title ,abstract,**  **keywords** | #1 orthodontic* OR "Tooth movement" OR "orthodontic tooth movement" OR "Tooth displacement " OR "orthodontic Treatment" OR "orthodontic Therapy"  #2 accelerat* OR rapid* OR short* OR speed* OR fast OR velocity OR duration OR rate OR time OR "regional accelerated phenomenon" OR RAP.  #3 (flapless (AND (corticotom* OR decorticat* OR surgery OR surgical OR alveolar surg* OR piezosurgery OR piezoelectric OR piezo*).  #4 (minimally invasive (AND (corticotom* OR decorticat* OR surgery OR surgical OR alveolar surg* OR piezosurgery OR piezoelectric OR piezo*).  #5 microsurgery OR micro incisions OR micro osteoperforations OR micro perforations OR perforations OR corticision OR lasercision OR corticopuncture OR piezocision OR piezotome OR piezopuncture.  #6 #3 OR #4 OR #5  #7 #1 AND #2 AND #6 |
| **EMBASE**  **Publication Date: from no limit Until 18-1-2016** | #1 orthodontic* OR "Tooth movement" OR "orthodontic tooth movement" OR "Tooth displacement " OR "orthodontic Treatment" OR "orthodontic Therapy"  #2 accelerat* OR rapid* OR short* OR speed* OR fast OR velocity OR duration OR rate OR time OR "regional accelerated phenomenon" OR RAP.  #3 (flapless( AND (corticotom* OR decorticat* OR surgery OR surgical OR alveolar surg* OR piezosurgery OR piezoelectric OR piezo*)  #4 ("minimally invasive") AND (corticotom* OR decorticat* OR surgery OR surgical OR alveolar surg* OR piezosurgery OR piezoelectric OR piezo*).  #5 microsurgery OR "micro incisions" OR "micro osteoperforations" OR "micro perforations" OR perforations OR corticision OR lasercision OR corticopuncture OR piezocision OR piezotome OR piezopuncture.  #6 #3 OR #4 OR #5  #7 #1 AND #2 AND #6 |
| **PubMed**  **Publication Date:**  **from no limit Until 18-1-2016 Search Builder:**  **All fields** | #1 orthodontic* OR "Tooth movement" OR "orthodontic tooth movement" OR "Tooth displacement " OR "orthodontic Treatment" OR "orthodontic Therapy"  #2 accelerat* OR rapid* OR short* OR speed* OR fast OR velocity OR duration OR rate OR time OR "regional accelerated phenomenon" OR RAP.  #3 (flapless (AND (corticotom* OR decorticat* OR surgery OR surgical OR alveolar surg* OR piezosurgery OR piezoelectric OR piezo*).  #4 (minimally invasive (AND (corticotom* OR decorticat* OR surgery OR surgical OR alveolar surg* OR piezosurgery OR piezoelectric OR piezo*).  #5 microsurgery OR micro incisions OR micro osteoperforations OR micro perforations OR perforations OR corticision OR lasercision OR corticopuncture OR piezocision OR piezotome OR piezopuncture.  #6 #3 OR #4 OR #5  #7 #1 AND #2 AND #6 |
| **Scopus**  Publication Date:  from no limit Until 18-1-2016 | #1TITLE-ABS-KEY (orthodontic* OR "Tooth movement" OR "orthodontic tooth movement” OR "Tooth displacement “OR "orthodontic Treatment” OR "orthodontic Therapy").  #2 TITLE-ABS-KEY(accelerat* OR rapid* OR short* OR speed* OR fast OR velocity OR duration OR rate OR time OR "regional accelerated phenomenon" OR RAP)  #3TITLE-ABS-KEY (flapless) ANDTITLE-ABS-KEY (corticotom*OR decorticat* OR surgery OR surgical OR alveolar surg* OR piezosurgery OR piezoelectric OR piezo*).  #4 TITLE-ABS-KEY ("minimally invasive") AND TITLE-ABS-KEY (corticotom* OR decorticat* OR surgery OR surgical OR alveolar surg* OR piezosurgery OR piezoelectric OR piezo*).  #5 TITLE-ABS-KEY (microsurgery OR "micro incisions" OR "micro osteoperforations" OR "micro perforations" OR perforations OR corticision OR lasercision OR corticopuncture OR piezocision OR piezotome OR piezopuncture *).*  #6 #3 OR #4 OR #5  #7 #1 AND #2 AND #6 |
| **Web of Science**  All databases  Publication Date:  from no limit Until 18-1-2016 | #1TS= (orthodontic OR "Tooth movement" OR "orthodontic tooth movement” OR "Tooth displacement “OR "orthodontic Treatment" OR "orthodontic Therapy").  #2TS= (accelerat* OR rapid* OR short* OR speed* OR fast OR velocity OR duration OR rate OR time OR "regional accelerated phenomenon" OR RAP).  #3TS= (flapless (AND TS= (corticotom* OR decorticat* OR surgery OR surgical OR alveolar surg* OR piezosurgery OR piezoelectric OR piezo*).  #4 TS= (minimally invasive (AND TS= (corticotom* OR decorticat* OR surgery OR surgical OR alveolar surg* OR piezosurgery OR piezoelectric OR piezo*).  #5TS= (microsurgery OR micro incisions OR micro osteoperforations OR micro perforations OR perforations OR corticision OR lasercision OR corticopuncture OR piezocision OR piezotome OR piezopuncture).  #6 #3 OR #4 OR #5  #7 #1 AND #2 AND #6 |
| **Google Scholar**  Publication Date:  from no limit Until 18-1-2016 | #1(orthodontic OR "Tooth movement") AND (accelerate OR acceleration OR accelerating OR accelerated OR rapid) AND (flapless corticotomy OR flapless decortication OR flapless surgery OR flapless piezosurgery )  #2 (orthodontic OR "Tooth movement") AND (accelerate OR acceleration OR accelerating OR accelerated OR rapid) AND (minimally invasive corticotomy OR minimally invasive decortication OR minimally invasive surgery OR minimally invasive piezosurgery )  #3 (orthodontic OR "Tooth movement") AND (accelerate OR rapid) AND (microsurgery OR micro incisions OR micro osteoperforations OR micro perforations OR corticision OR corticopuncture OR piezocision OR piezotome OR piezopuncture). |
| **Trip**  Publication Date:  from no limit Until 18-1-2016 | )orthodontic OR "Tooth movement" OR "orthodontic tooth movement" OR "Tooth displacement " OR "orthodontic Treatment" OR "orthodontic Therapy") AND (accelerate OR acceleration OR accelerating OR accelerated OR rapid OR short OR speed OR fast OR velocity OR duration OR rate OR time OR "regional accelerated phenomenon" OR RAP) AND( flapless corticotomy OR flapless decortication OR flapless surgery OR flapless piezosurgery OR minimally invasive corticotomy OR minimally invasive surgery OR minimally invasive surgical OR microsurgery OR micro incisions OR micro osteoperforations OR micro perforations OR perforations OR corticision OR lasercision OR corticopuncture OR piezocision OR piezotome OR piezopuncture). |
| **OpenGrey**  <http://www.opengrey.eu/> | #1 acceleration AND tooth movement  #2 orthodontic AND acceleration  #3 flapless corticotomy OR flapless decortication OR minimally invasive corticotomy OR micro osteoperforations OR corticision OR lasercision OR corticopuncture OR piezocision OR piezosurgery OR piezoelectric surgery OR flapless piezoelectric corticotomy OR piezotome OR piezopuncture . |
| **PQDT OPEN** **(from proQuest)** <http://pqdtopen.proquest.com/> | #1(orthodontic OR "Tooth movement") AND (accelerate OR acceleration OR accelerating OR accelerated OR rapid) AND (flapless corticotomy OR flapless decortication OR flapless surgery OR flapless piezosurgery )  #2(orthodontic OR "Tooth movement") AND (accelerate OR acceleration OR accelerating OR accelerated OR rapid) AND (minimally invasive corticotomy OR minimally invasive decortication OR minimally invasive surgery OR minimally invasive piezosurgery).  #3 (orthodontic OR "Tooth movement") AND (accelerate OR rapid) AND (microsurgery OR micro incisions OR micro osteoperforations OR micro perforations OR corticision OR lasercision OR corticopuncture OR piezocision OR piezotome OR piezopuncture) |
