# Supplementary material for: Effectiveness of minimally invasive surgical procedures in the acceleration of tooth movement: a systematic review and meta-analysis
Source: Prog Orthod. 2016 Oct 24;17:33. doi: 10.1186/s40510-016-0146-9 (PMC5075528; doi:10.1186/s40510-016-0146-9)
Supplement: Additional file 2: Table S2. — Search strategy for trials’ registries. (DOCX 13 kb) [file 40510_2016_146_MOESM2_ESM.docx]

| **Table S2: Search strategy for trials' registries** | |
| --- | --- |
|  | **Search terms** |
| **ClinicalTrials.gov**  **(Last updated 18-1-2016)** | Orthodontic AND acceleration , Orthodontic AND accelerating, Orthodontic AND accelerate ,Tooth movement AND accelerated , Flapless corticotomy, Flapless decortication, Flapless piezosurgery, Flapless piezoelectric, Piezocison, Corticision, Micro-osteoperforations , Minimally invasive AND Corticotomy, Minimally invasive AND accelerated , minimally invasive AND acceleration , Minimally invasive AND accelerating, Minimally invasive AND orthodontics , Rapid orthodontics . |
| **World Health Organization International Clinical Trials Registry Platform Search Porta**l (**ICTRP**)  **(Last updated 18-1-2016)** | Orthodontic AND acceleration , Orthodontic AND accelerating, Orthodontic AND accelerate , Tooth movement AND accelerated , Flapless corticotomy, Flapless decortication, Flapless piezosurgery, Flapless piezoelectric, Piezocison, Corticision, Micro-osteoperforations , Minimally invasive AND corticotomy, Minimally invasive AND accelerated ,Minimally invasive AND acceleration , Minimally invasive AND accelerating, Minimally invasive AND orthodontics , Rapid orthodontics. |
