# Supplementary material for: Effectiveness of minimally invasive surgical procedures in the acceleration of tooth movement: a systematic review and meta-analysis
Source: Prog Orthod. 2016 Oct 24;17:33. doi: 10.1186/s40510-016-0146-9 (PMC5075528; doi:10.1186/s40510-016-0146-9)
Supplement: Additional file 3: Table S3. — Excluded studies and the reasons beyond exclusion. (DOCX 16 kb) [file 40510_2016_146_MOESM3_ESM.docx]

| **Table S3: Excluded studies and the reasons beyond exclusion** | |
| --- | --- |
| **Study** | **Reason for exclusion** |
| Milano, F., Dibart, S., Montesani, L. and Guerra, L. (2014) Computer-guided surgery using the piezocision technique. *The* *International og Periodontics and Restorative Dentistry*, 34, 523-529. | Case report |
| Cassetta, M., Pandolfi, S. and Giansanti, M. (2015) Minimally invasive corticotomy in orthodontics: a new technique using a CAD/CAM surgical template*.* *International Journal of Oral and Maxillofacial Surgery*, 44, 830-833. | Case report |
| Keser, E.I. and Dibart,s. (2013) Sequential piezocision: a novel approach to  accelerated orthodontic treatment*.* *American Journal of Orthodontics and*  *Dentofacial Orthopedics* ,144, 879-889. | Case report |
| Faraji, M., Camacho, M.A.I., Fernández, S.T., Ledesma, A.F. and Hernández, C. (2014) Tratamiento ortodóncico acelerado periodontalmente: comparación de técnicas quirúrgicas*.* *Revista Mexicana de Periodontologia*, 1, 30-35. | Absence of a control group |
| Ileri, Z., Akin, M., Erdur, E.A., Dagi, H.T. and Findik, D. (2014) Bacteremia after piezocision*. American Journal of Orthodontics and Dentofacial Orthopedics* , 146, 430-436. | Not a randomized trial (i.e. a cohort study): “Thirty subjects 24 women, 6 men; average age 19.6+_60.7 years with the American Society of Anesthesiologists' physical status I and mild to moderate crowding who were scheduled to have nonextraction treatment were enrolled in this study” |
| Salman, L.H. and Ali, F.A. (2014) Acceleration of canine movement by laser assisred flapless corticotomy [An innovative approach in clinical orthodontics]. *Journal of of Baghdad College of Dentistry*, 26,133-137. | Not a randomized trial. The study was a split-mouth design and the side subjected to surgery was selected by the surgeon and not by pure chance. The surgeon chose the side “with more space between the canine and second premolar” |
| Makhoul, F., Jaafo, M.H. and Ajai, M. (2014) Evaluation of the effectiveness of Alveolar Corticotomy on reducing orthodontic root resorption. *Journal of Indian Dental Association,* 8, 31-39 | Not a randomized trial but rather a controlled clinical trial (CCT). The surgical intervention was invasive despite the use of a flapless corticotomy since the authors employed a tunneling technique for alveolar bone surgery and sutures were needed to close the surgical wounds. |
| Alikhani, M., Alansari, S., Sangsuwon, C., Alikhani, M., Chou, M.Y., Alyami, B., Nervina, J.M. and Teixeira, C.C. (2015) Micro-osteoperforations: Minimally invasive accelerated tooth movement. *Seminars in Orthodontics*, 21,162-169. | Although the general type appeared to be a randomized controlled trial but the study methodology and the reporting of outcomes were very poor. Many important data were missing: age range, gender distribution, technical aspects of interventions, actual values of differences between the experimental and control groups. The authors were contacted for clarifications but with no response (see supplementary table 3). |
| NCT01093352: Effect of Alveolar-decortication on Velocity of Tooth Movement | Ongoing trial (protocol): invasive surgery (flap elevation: impacted canines) |
| IRCT2013082014415N1: The effect of buccal corticotomy in accelerating orthodontic canine movement | Ongoing trial (protocol): invasive surgery (flap elevation) |
| NCT02026258: Efficiency of Piezotome-Corticision Assisted Orthodontics | Registered Protocol for a completed trial (Mehr 2013) which was already been included in the systematic review. |
