# Supplementary material for: Effectiveness of minimally invasive surgical procedures in the acceleration of tooth movement: a systematic review and meta-analysis
Source: Prog Orthod. 2016 Oct 24;17:33. doi: 10.1186/s40510-016-0146-9 (PMC5075528; doi:10.1186/s40510-016-0146-9)
Supplement: Additional file 4: Table S4. — (Supplemental to Table 1): Additional Characteristics of the included studies. (DOCX 16 kb) [file 40510_2016_146_MOESM4_ESM.docx]

| **Table S4 (Supplemental to table 1): Additional Characteristics of the included studies** | | | | | | |
| --- | --- | --- | --- | --- | --- | --- |
| **Study** | **Appliance characteristics + Biomechanics** | **Anchorage used** | **Frequency of orthodontic**  **adjustments** | **Post - treatment drugs** | **Statistical significance of reported difference treated vs controls** | **Methods of primary outcome measurements** |
| Alikhani  2013  [17] | -0,022 inch slot MBT brackets. -0.016 x 0.022-inch SS archwire  -U3 retraction achieved with (100 gm) NiTi coil springs connected from a TAD to a power arm on the canine bracket. | TADs was placed between the second premolar and the first molar | NR | NO post intervention drugs were prescribed | ***RTM (mm/month) after 28 days :*** P-value < 0,05 Exp: 1,15+-0,15 , control: 0,51+-0,15 Acceleration rate: 2,3 Times  **Pain and discomfort** :( P-value > 0,05)  Using Numeric Rating Scale (NRS) 1d, 7d, and 28 days after retraction.  **Inflammatory markers (Cytokines levels):**  (P-value<0,05) in gingival cervicular fluid was collected before retraction, 1d, 7d, and 28 days after retraction. | Study models using an electric digital calipers |
| Mehr  2013  [38] | -0,022 inch slot self-ligating brackets  -0,014 Cu-NiTi: first 2 visits -0,014 x 0,025 Cu-NiTi | Not Reported | Every 4 to 5 weeks | Clorhexidine  Acetaminophen  (if needed) | ***RTM (mm/month)***: 1^st^ month: P-value =0,035 Exp: -0,109+_0,033 , control:- 0,068+-0,025 2^nd^ ,3^rd^ ,4^th^  months : P-value > 0,05  2^nd :^  Exp: -0,061+_0,032 , control: -0,073+_0,061  3^rd:^  Exp: -0,023+_0,022 , control: -0,068+_0,059  4^th:^ Exp: -0,025+-0,002 , control: -0,037+_0,004 Acceleration rate: 1.6 times in only the first 4-5 weeks  ***TTM (days):*** P-value = 0,43 Exp:98,50+-30,38 , control:118,40+_40,77  **Pain score**: using Visual Analog Scale(VAS) : (p>0,05) 1h, 12h, 7days | Study models using digital calipers |
| Leethanakul 2014  [39] | -Roth’s prescription pre-adjusted edgewise brackets  -power arm 0.021 x0.025 SS  -Lingual buttons on canines and molars. -Elastomeric chains (150 g) | TADs were placed between the second premolars and first molar | Every 4 weeks | NR | ***RTM (mm/month)***: 1^st ,^ 2^nd^  month: P-value =0.002 1^st:^  Exp:1.60+_1.08 , control:0.9+_0.3 2^nd:^  Exp:2.3+_1.1 , control:1.2+_0.5 3^rd^ month: P-value > 0.005 Exp:1.6+_0.8 , control:1.3+_0.7  ***CTM (mm):*** 1^st^  month^:^  P-value =0.002  Exp:1.6+_1.08, control:0.9+_0.3 2^nd^ month^:^  P-value =0.003  Exp:3.9+_1.8 ***,*** control:2.1+_0.9  3^rd^  month^:^ P-value =0.002  Exp:5.4+_1.5 ***,*** control:3.4+_0.9  **Canine tipping:** (p-value > 0,05) using lateral cephalograms .  **Canine rotation:** (P-value > 0,05) using Study models, | Study models |
| Aksakalli 2015  [40] | -0.022-inch slot Roth’s brackets.  -0.016 x 0.022-inch SS archwire The distalization was performed using elastomeric chains with an approximate force of 150 g. | Moderate anchorage  (Not reported the method) | Every 2 weeks  (the elastomeric chains were replaced at each appointment) | NR | ***CTM (mm):*** ( P-value<0,05)  1^st^  month^:^ Exp:1.53+_ 0.67 , control: 0.78 +_ 0.24 2^nd^ month^:^ Exp: 2.90 +_ 0.86 , control: 1.73 +_ 0.72  ***TTM (months):*** ( P-value<0,05)  Exp: 3.54 +_ 0.81  control: 5.59 +- 0.94  **molar Anchorage Loss (mm) :**  ( P-value<0,05) using 3D models, anchorage loss was lesser after piezocision  **Transversal changes:** ( P-value > 0,05) using 3D models **mobility scores for the canines:**  (P-value > 0,05) using Muhleman’s index  **gingival indices for the canines:**  ( P-value > 0,05) using Silness and Loe | model casts were scanned using the 3Shape R900 scanner |
| **RTM: Rate of tooth movement; TTM: Time of tooth movement; CTM: Cumulative tooth movement; EXP: Experimental; NR: Not reported; TADs: Temporary Anchorage Devices; U3: upper canines; SS: Stainless Steel** | | | | | | |
