# Supplementary material for: Effectiveness of minimally invasive surgical procedures in the acceleration of tooth movement: a systematic review and meta-analysis
Source: Prog Orthod. 2016 Oct 24;17:33. doi: 10.1186/s40510-016-0146-9 (PMC5075528; doi:10.1186/s40510-016-0146-9)
Supplement: Additional file 5: Table S5. — (Supplementary to Table 2): Additional Characteristics of the protocols (ongoing studies). (DOCX 15 kb) [file 40510_2016_146_MOESM5_ESM.docx]

| **Table S5 (Supplementary to table 2): Additional Characteristics of the protocols (ongoing studies)** | | | | |
| --- | --- | --- | --- | --- |
| **Study *ID*** | **Setting** | **Orthodontic aspects** | **Technical aspects of interventions** | **Notes** |
| NCT02606331 | Orthodontic Department, University of Damascus Dental School | -U3 retraction will be begun after completion of the leveling and alignment using 150 gm NiTi closed coil springs  -Anchorage used: soldered trans-palatal arch  Baseline Characteristics: Class II Division 1 malocclusion | NR | This study is currently recruiting participants  Starting date: April 2015  Completion date: June 2016 |
| NCT02359760 | University of Montreal, orthodontic department | The appointments will be every 2 weeks for 4 months, then every month  Baseline Characteristics: Class I with" Irregularity index" of < 4mm | Piezocision one week after bracket placement | This study is not yet open for participant recruitment  Starting date: February 2015  Completion date: May 2017 |
| NCT02590835 | University Hospital of Liege | - Damon self-ligating systems.  -0.014-inch copper-nickel-titanium Damon arch forms.  -The appointments will be every two weeks.  -The sequence of archwires was as follows: 0.018-in, 0.014x0.025-in and 0.018x0.025-in copper nickel-titanium archwires for alignment; and 0.019x0.025-in stainless steel archwires for fine-tuning.  Baseline Characteristics: Tooth Overcrowding. | Piezocision was performed one week after orthodontic appliance placement, vertical interproximal incisions were performed below each interdental papilla, 5 mm long and 3 mm deep and no sutures were required. | This study has been completed  Starting date: February 2013  Completion date: October 2015 |
| NCT01720797 | University of Florida, Department of Orthodontics | The appointments will be every four to six weeks, after completion the first follow-up.  Baseline Characteristics: NR | NR | This study has been completed  Starting date: February 2013  Completion date: April 2015 |
| NCT02549950 | King Abdullah International Medical Research Center | canine and incisor retraction  Baseline Characteristics: Angle Class II Division 1 , bi-maxillary protrusive | Peizo-Corticision:  Part I: retraction of canine teeth in class II div 1 subjects. Part II: retraction of anterior teeth in class II div 1 and class 1 bi-maxillary protrusive subjects. | This study is currently recruiting participants  Starting date: September 2015  Completion date: July 2017 |
| NCT02473471 | Jordan University of science and technology | NR  Baseline Characteristics:  Class II Division 1 malocclusion | Three small MOPs will be performed distal to canine in cortical bone by Mini screws. | This study is currently recruiting participants  Starting date: March 2015  Completion date: May 2017 |
| NCT02571348 | Faculty of Dentistry, University of Malaya ,Malaysia | Baseline Characteristics: NR  -Molar Class I malocclusion, molar Class II & III < ½ unit. -requiring extraction of all four permanent first premolar  -temporary anchorage device (TAD) for maximum anchorage . | MOPs will be performed at 4, 8 and 12 weeks intervals, by mini implant. | This study is currently recruiting participants  Starting date: September 2015  Completion date: June 2017 |
| NCT02416297 | University of Sao Paulo , Brazil | Retraction of anterior teeth using Light orthodontic force will be performed to close extraction space.  Baseline Characteristics: Tooth extraction indication for orthodontic treatment. | Two MOPs will be performed by manual instrument after first premolars extraction, 4-6 mm below the interdental papilla between the canine and second premolar. | This study is currently recruiting participants  Starting date: April 2015  Completion date: July 2017 |
| ACTRN12615000593538 Register: ANZCTR | Australia Department of Orthodontics, Sydney Dental Hospital | Nickel Titanium springs used to retract canines  Baseline Characteristics:  Patients who need to upper premolar extractions. (Maxillary protrusion or bimaxillary protrusive and severe crowding). | Three MOPs will be performed by disposable appliance designed for this purpose by Propel Orthodontics, on the mesial and distal aspects of the upper first premolar. | Recruitment status: Not yet recruiting  Starting date: July 2015  Completion date: NR |
| **MOPs: Micro osteo-perforations; NR: not reported; U3: upper canines;** | | | | |
