# Supplementary material for: Effectiveness of minimally invasive surgical procedures in the acceleration of tooth movement: a systematic review and meta-analysis
Source: Prog Orthod. 2016 Oct 24;17:33. doi: 10.1186/s40510-016-0146-9 (PMC5075528; doi:10.1186/s40510-016-0146-9)
Supplement: Additional file 6: Table S6. — Risk of bias. (DOCX 16 kb) [file 40510_2016_146_MOESM6_ESM.docx]

| **Table S6: Risk of bias** | | | | | | | |
| --- | --- | --- | --- | --- | --- | --- | --- |
| **Study** | **sequence generation** | **Allocation concealment** | **Blinding of participants and personnel** | **Blinding of outcome assessment** | **Incomplete outcome data** | **Selective reporting** | **Other bias** |
| Alikhani  2013  [17] | Unclear risk:  No mention of the method used for randomisation “patients were randomly assigned to one of the study groups” (Page 640) ,  Authors were emailed for explanation but there was no response. | Unclear risk:  no mention of the method used to conceal the allocation sequence ,authors were emailed for explanation but there was no response | Unclear risk:  Blinding cannot be performed “The subjects and the residents administering the treatment were aware of the group assignment and therefore were not blinded.” (Page 640), it is not clear if non-blinding could affect the outcomes. | Low risk:  “The investigators performing the measurements and data analysis were blinded from the group assignments.” (Page 640), it was possibly done. | Low risk:  No dropouts were reported | Low risk:  The protocol was not registered. But the pre-defined outcomes that mentioned in the methods section seemed to have been reported. | Unclear risk:  Due to a possible conflict of interest Propel Orthodontics Inc. licensed and developed a tool for the procedure (MOPs) but did not support the study. NYU buy the Propel tools used in this trial. It is unclear whether this would affect the outcome. |
| Mehr  2013  [38] | Low risk:  “Randomization sequences were generated using random block sizes of six and eight and allocation ratio of 1:1 with the Random Allocation Software program” (page 16) , it was possibly done. | Low risk:  “The allocation sequences were sealed around aluminum foil in envelopes with identical appearance, and were stored in a box. Once patients were enrolled in the study, the study coordinator (RM) picked and opened the envelopes sequentially.” Page 16 وprobably done. | Unclear risk:  No details of blinding of participant.  We emailed authors for explanation, but the risk of selection bias was still unclear. | Low risk:  “Patient codes were assigned to the models prior to measurement to ensure blinding.” (Page 20), it was possibly done. | Low risk: “Out of the 14 patients enrolled in the study, 1 control patient did not receive any intervention due to a change of the treatment plan and 1 experimental patient was lost to follow-up“ (page 34) , incomplete outcome data adequately. Addressed (number , reasons) | Low risk:  The protocol for the study was registered in clinical trial.gov study ID:  (NCT02026258) and the outcomes that mentioned in the protocol have been reported | Low risk:  No other forms of bias seemed to be found. |
| Leethanaku**l**  2014  [39] | Low risk: No reporting of the method used for randomisation, authors were emailed for explanation: “We allocated the experimental side from a pile of pre-shuffled cards. By order of entry, the card on the top of the pile would be opened to designate the experimental side of the subject.” it was possibly done. | Low risk:  NO mention of the method used to conceal the allocation sequence, authors were emailed for explanation: “The surgeons were told by the researcher which side was to be experimental side. After that, the allocation data was concealed. The orthodontists treated the subject without knowing which side was experimental side.” it was possibly done. | Low risk:  No information of blinding is mentioned ,  Authors were emailed for explanation. “The surgeons were told by the researcher which side was to be experimental side. After that, the allocation data was concealed. The orthodontists treated the subject without knowing which side was experimental side”, it was possibly done. | Unclear risk: No details of blinding of outcome assessors.  Authors were emailed for explanation, but the risk of bias was still unclear. | Low risk:  No dropouts were reported in the paper.  Authors were emailed for explanation: ” 3 samples excluded  because the alveolar bone width of the first bicuspid was narrower than the root of canine” incomplete outcome data adequately addressed (numbers , reasons ) | Low risk:  The protocol was not registered. But the pre-defined outcomes that mentioned in the methods section seemed to have been reported. | Low risk:  No other forms of bias seemed to be found. |
| Aksakalli  2015  [40] | Low risk:  No details about randomization method. Authors were emailed for explanation : “We used computer programme to randomize patients”, it was possibly done | Unclear risk:  No information about allocation concealment Authors were emailed for explanation but the risk of bias was still unclear. | Unclear risk:  Blinding of participants and personnel was not feasible, authors were emailed for explanation: “it is not possible to blind piezocision application stage” it is not clear if non-blinding could affect the outcomes. | Low risk:  No mention of blinding of outcome assessors, authors were emailed for explanation: “there was blinding in every applicable stage such as 3D measurements and statistics.” it was possibly done | Low risk:  No dropouts were reported. | Low risk:  The protocol for the study was not registered, but the predefined outcomes that mentioned in the methods section seemed to have been reported | Low risk:  No other forms of bias seemed to be found. |
